# Investigation of Transovarial Transmission of *Bartonella henselae* in *Rhipicephalus sanguineus* sensu lato Ticks Using Artificial Feeding

**DOI:** 10.3390/microorganisms9122501

**Published:** 2021-12-02

**Authors:** Wittawat Wechtaisong, Sarah I. Bonnet, Bruno B. Chomel, Yi-Yang Lien, Shih-Te Chuang, Yi-Lun Tsai

**Affiliations:** 1 Department of Veterinary Medicine, College of Veterinary Medicine, National Pingtung University of Science and Technology, Pingtung 912, Taiwan; wittwech@gmail.com (W.W.); yylien@mail.npust.edu.tw (Y.-Y.L.); 2 Animal Health Department, INRAE, 37380 Nouzilly, France; sarah.bonnet@inrae.fr; 3 Functional Genetics of Infectious Diseases Unit, Institut Pasteur, CNRS UMR 2000, Université de Paris, 75015 Paris, France; 4 Department of Population Health and Reproduction, School of Veterinary Medicine, University of California, Davis, CA 95616, USA; bbchomel@ucdavis.edu; 5 Department of Veterinary Medicine, School of Veterinary Medicine, National Chung Hsing University, Taichung 402, Taiwan; stchuang@dragon.nchu.edu.tw

**Keywords:** transovarial transmission, *Bartonella henselae*, *Rhipicephalus sanguineus* sensu lato

## Abstract

*Bartonella henselae* is a slow-growing, Gram-negative bacterium that causes cat scratch disease in humans. A transstadial transmission of the bacteria from larvae to nymphs of *Rhipicephalus sanguineus* sensu lato (s.l.) ticks, suspected to be a potential vector of the bacteria, has been previously demonstrated. The present study aims to investigate transovarial transmission of *B. henselae* from *R. sanguineus* s.l. adults to their instars. Adult ticks (25 males and 25 females) were fed through an artificial feeding system on *B. henselae*-infected goat blood for 14 days, and 300 larvae derived from the experimentally *B. henselae*-infected females were fed on noninfected goat blood for 7 days. Nested PCR and culture were used to detect and isolate *B. henselae* in ticks and blood samples. *Bartonella henselae* DNA was detected in midguts, salivary glands, and carcasses of the semi-engorged adults and pooled tick feces (during feeding and post-feeding periods). After the oviposition period, *B. henselae* DNA was detected in salivary glands of females (33.3%), but not in pooled eggs or larvae derived from the infected females. However, *B. henselae* DNA was detected by nested PCR from the blood sample during larval feeding, while no viable *B. henselae* was isolated by culture. According to our findings, following infected blood meal, *B. henselae* could remain in the tick midguts, move to other tissues including salivary glands, and then be shed through tick feces with limited persistency. The presence of bacterial DNA in the blood during larval feeding shows the possibility of transovarial transmission of *B. henselae* in *R. sanguineus* s.l. ticks.

## 1. Introduction

*Bartonella henselae* is a slow-growing, intraerythrocytic Gram-negative bacterium that infects humans and companion animals [1]. Cat fleas (*Ctenocephalides felis*) are known to be the vector of the bacterium from cat to cat [2,3,4]. The bacterium can remain in the gut of cat fleas for up to 9 days and be excreted with flea feces [5]. *Bartonella* *henselae* is usually transmitted to humans by a cat scratch contaminated with flea feces and causes cat-scratch disease (CSD) [6]. The disease is self-limiting from asymptomatic to fever, skin inflammation, and lymphadenopathy in most human cases [7]. However, bacillary peliosis and angiomatosis can occur in immunocompromised patients [8,9].

Viable *B. henselae* or its DNA has also been isolated or detected in several other blood-feeding arthropods, such as biting flies, keds, lice, and ticks, which indicates the wide range of potential vectors that may transmit *B. henselae* to humans or companion animals [10,11,12,13,14,15,16,17]. Ticks have been suspected of being a potential vector of *Bartonella* spp. since 1992, when *Bartonella* spp. were isolated from the blood of patients following tick bites [18]. Both transstadial transmission of *B. henselae* from larvae to nymphs and from nymphs to adults and transmission to the blood via saliva of ticks infected at the preceding stage have been experimentally demonstrated for *Ixodes ricinus* ticks [19]. Moreover, *I. ricinus* ticks can also maintain *B. birtlesii* from larvae to nymphs and from nymphs to adults, and the ticks infected with *B. birtlesii* at the preceding life stages can transmit the bacteria to naïve mice [20]. These results demonstrated that *I. ricinus* is a competent vector for both *B. henselae* and *B. birtlesii* [19,20]. More recently, *B. henselae*, *B. grahamii*, and *B. schoenbuchensis* DNA were found across the developmental stages of experimentally infected *I. ricinus*, suggesting more evidence on the potential *Bartonella* transmission by ticks [21].

*Rhipicephalus sanguineus* s.l. is a three-host ixodid tick, which mainly parasitizes dogs [22]. *Rhipicephalus sanguineus* s.l. needs a new host to feed on for each developmental stage [22]. This tick has a worldwide geographical distribution and is a vector of several pathogens causing clinical illnesses in humans and companion animals [23]. *Bartonella henselae* DNA has been detected in *R. sanguineus* s.l. ticks thanks to epidemiological surveys performed in several parts of the world [17,24,25]. Our previous study revealed the possibility of transstadial transmission of *B. henselae* from *R. sanguineus* s.l. larvae to nymphs [26]. After feeding with *B. henselae*-infected blood, *B. henselae* DNA was detected in engorged larvae, semi-engorged larvae, and larval feces, suggesting *Bartonella* acquisition by ticks. Nymphs molted from the infected larvae also harbored *B. henselae* DNA and were able to transfer bacterial DNA into blood through saliva [26].

The present study aimed to validate the *B. henselae* infection in *R. sanguineus* s.l. adults and transmission of the bacteria to their instars using an artificial membrane feeding system. Validating such a transovarial transmission can provide additional evidence regarding the fact that *R. sanguineus* s.l. could be a potential vector for *B. henselae*.

## 2. Materials and Methods

### 2.1. Study Design

**Figure 1:**
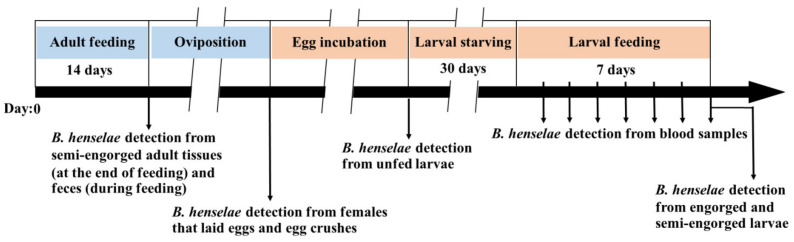
Experimental framework of *R. sanguineus* s.l. adult ticks infected by *B. henselae*-infected blood and bacteria DNA detection in tick and blood samples.

The experimental framework is shown in Figure 1. Fifty *R. sanguineus* s.l. adult ticks were fed with *B. henselae*-infected goat blood for 14 days and allowed to complete their oviposition period. After the period of egg hatching and emergence of larvae, 300 larvae were starved for 30 days and then fed with noninfected goat blood for 7 days. As a control group, another 40 adult ticks and 250 larvae were fed with noninfected goat blood, respectively. Nested PCR was used to detect *B. henselae* DNA from semi-engorged adults (at the end of adult feeding), pooled tick feces (collected daily during adult feeding and post feeding), females and egg samples (post oviposition), unfed larvae (post hatching), blood samples (collected daily during larval feeding), and engorged larvae (at the end of larval feeding). Tick feces at the end of adult feeding and blood samples collected daily during larval feeding were also used for *B. henselae* isolation by culture.

### 2.2. Rhipicephalus sanguineus s.l. Ticks

*Rhipicephalus sanguineus* s.l. ticks from *B. henselae*-negative batches were reared at room temperature, 80–90% relative humidity, and a photoperiod of 16:8 h (L:D) [27]. The tick colonies were maintained by feeding on an artificial feeding system, and the duration of feeding was 7 days for larvae, 10 days for nymphs, and 14 days for adults [27,28].

### 2.3. Bartonella henselae Isolates

*Bartonella henselae* isolates were obtained from a stray cat in Taitung, Taiwan. The cat blood was cultured on chocolate agar plates (Taiwan Prepared Media Co., Ltd, Taipei, Taiwan), and incubated at 35 °C, 5% CO_2_ for 14 days. After being confirmed by nested PCR, *B. henselae* colonies were used for tick infection and as a positive control for the PCR assay. The number of viable bacteria was estimated by colony-forming unit (CFU). For tick infection, *B. henselae* colonies were harvested from agar plates by using a sterilized polypropylene loop (SPL Lifesciences Co., Ltd., Gyeonggi-do, Korea), suspended in sterile 1× phosphate-buffered saline (1× PBS), and used immediately for tick infection [19].

### 2.4. Artificial Membrane Feeding System

An artificial membrane feeding system with mouse skin was adapted from Bonnet et al. [28] and successfully used in a previous study for *R. sanguineus* s.l. larval and nymphal feeding [26]. The feeding system consists of a glass feeder, tick container, and skin membrane. Mouse skins were dissected, sterilized, and aseptically treated by previously described processes [26,28]. Goat blood, confirmed *Bartonella* DNA-negative by PCR, was used to feed ticks in all experiments. The blood was collected from goats in Pingtung, Taiwan (ethical permit IACUC number: NPUST-108-066), before being subjected to defibrination and functional complement depletion by heat treatment at 56 °C for 30 min [19]. Before tick feeding, decomplemented blood was supplemented with fosfomycin (20 µL/mL), amphotericin B (0.25 µg/mL), and heparin (10 kU/mL) [19].

### 2.5. B. henselae Infection in R. sanguineus s.l. Adult Ticks

A total of 25 male and 25 female adult ticks were placed in the tick container of the feeding system and fed on *B. henselae*-infected blood for 14 days with the same laboratory conditions as previously described [28]. Briefly, 6 mL of decomplemented goat blood was mixed with 6 µL of *B. henselae* suspension at the concentration of 10^9^ CFU/mL and added to the artificial feeder for tick infection. The feeding system was connected with a 37 °C water circulation to mimic host body temperature, attract ticks, and maintain *B. henselae* in the blood. *Bartonella henselae*-infected blood was renewed every 12 h after washing the mouse skin three times with RPMI 1640 (Corning Inc., Corning, NY, USA) [28]. In addition, adult ticks were manually detached from mouse skins before the skin replacement, which was renewed every 5 days until the end of the feeding, and the ticks were then allowed to reattach to the skins to continue their blood meal. As a control group, 20 males and 20 females were also fed on noninfected goat blood with the same laboratory conditions, and the blood was renewed once a day.

During the feeding experiment, 19 samples of pooled adult tick feces were collected from tick containers using a sterilized polypropylene loop and needle (SPL Lifesciences Co.,Ltd., Gyeonggi-do, Korea): twice on day 1 (12 h and 24 h), once per day from day 2 to day 13, and five times on day 14. The 15 samples collected from day 1 to day 14 were suspended in 100 µL of sterile 1× PBS for DNA extraction, and the remaining four fecal samples collected on day 14 were incubated at room temperature (24 °C, 60% humidity) for 1, 3, 7, and 10 days, respectively. At the end of each incubation period, each fecal sample was suspended in 200 µL of 1× PBS and used as follows: 100 µL was used to detect *B. henselae* DNA by nested PCR, and 100 µL was cultured for *B. henselae* isolation.

After 14 days of feeding, in both experimental and control groups, semi-engorged ticks were manually detached and kept in 75% ethanol until the PCR detection for *B. henselae*. The ticks engorged until repletion were then individually placed in separate containers and allowed to lay eggs. After the oviposition period, the female ticks and 15–20 of the eggs laid by each female were then individually tested for *B. henselae* DNA presence by nested PCR.

### 2.6. Feeding Larval Ticks with Non-Infected Blood

After 1 month of starving, 300 larvae derived from three *B. henselae*-infected engorged females (around 100 larvae from each batch) were fed on noninfected goat blood for 7 days with the similar laboratory conditions used for adults. For the control group, 200 larvae from engorged females fed on noninfected blood were also fed in another feeder. During larval feeding, the blood sample was collected daily from each feeder before blood renewing, and 200 µL and 100 µL of the collected blood were used to detect *B. henselae* DNA by nested PCR and by culture, respectively.

### 2.7. DNA Extraction from Tick Samples, Tick Feces, Blood Samples, and Bacterial Colonies

Thirteen kinds of samples were used for *B. henselae* detection, including midguts, salivary glands, and carcasses from male ticks (at the end of adult feeding), midguts, salivary glands, ovaries, and carcasses from female ticks (at the end of adult feeding and post-oviposition), pooled adult feces from tick containers (during adult feeding), pooled 15 laid eggs, pooled 15 unfed larvae, pooled 5 engorged larvae, 100 µL of blood samples, and bacterial colonies.

Tick samples from both females and males were dissected in cold 1× PBS (4 °C). All dissection materials were cleaned with NucleoClean Decontamination spray (Merck Millipore, Burlington, MA, USA) before the dissection process. Each tissue and organ was rinsed with sterile distilled water before placed individually in a microcentrifuge tube [29]. The rest of tick body, i.e., the tick carcass, was placed in another microcentrifuge tube. A total of 100 µL of sterile 1× PBS was then added to each tube and stored at 4 °C until the DNA extraction process.

Bacterial colonies cultured on chocolate agar plates (see below) were randomly collected by using sterilized polypropylene loop (SPL Lifesciences Co., Ltd., Pocheon, Korea), suspended in 100 µL of 1× PBS and stored at 4 °C until DNA extraction process.

DNA from ticks, tick feces, blood samples, and bacterial colonies was extracted using the DNeasy Blood and Tissue kit (Qiagen, Hilden, Germany) following the manufacturer’s instructions. All DNA samples were eluted into a 50 µL final volume and stored at −20 °C until used for *B. henselae* detection by nested PCR.

### 2.8. Nested PCR Assay

The nested PCR was performed in a LabCycler (Sensoquest, Gottingen, Germany). The primers, forward 5′–CTTCGTTTCTCTTTCTTCA–3′ and reverse 5′–CTTCTCTTCACAATTTCAAT–3′, were used for the outer reaction to amplify a 472 bp segment of the 16S–23S rRNA ITS region of *Bartonella* spp. [30]. The primers, forward 5′–TTGCTTCTAAAAAGCTTATCAA–3′ and reverse 5′–CAAAAGAGGGATTACAAAATC–3′, were used for the inner reaction to amplify a 254 bp segment of *B. henselae* [31]. PCR mixture was carried out in a 50 µL reaction volume which contained 5 µL of DNA template, 1 µL of 10 µM of each primer, 5 µL of 10× *Taq* buffer (Genomics BioSci & Tech, Taipei, Taiwan), 4 µL of 2.5 mM of dNTP mixture (Genomics BioSci & Tech, Taipei, Taiwan), and 1 µL of 2.5 U/µL of *Taq* DNA polymerase (Genomics BioSci & Tech, Taipei, Taiwan), adjusted to the final volume with distilled water. DNA from a single colony of *B. henselae* (positive control) and distilled water (negative control) were used as controls for the PCR assay. The PCR conditions for both outer and inner reactions were those described by Sato et al. [31]. The expected PCR products (254 bp segment) were separated using a 2% agarose gel stained with nucleic acid stain (HealthView, Genomics BioSci & Tech, Taipei, Taiwan), and the results were visualized with UVIdoc HD5 (Uvitec, Cambridge, UK).

### 2.9. Culturing of B. henselae from Blood during Larval Feeding and Tick Fecal Samples

To isolate viable *B. henselae*, 100 µL of blood from feeders was placed in 1 mL of Schneider *Drosophila* medium (Thermo Fisher Scientific Taiwan Co., Ltd., Kaohsiung, Taiwan) and incubated at 35 °C, 5% CO_2_ for 6 days [19]. Then, 100 µL of the incubated sample was placed on chocolate agar plates (Taiwan Prepared Media Co., Ltd., Taipei, Taiwan) and incubated under the same conditions for 1 month. For *B. henselae* isolation from pooled adult tick feces, 100 µL of suspended pooled tick feces was directly placed on chocolate agar plates and incubated at 35 °C, 5% CO_2_ for 1 month.

### 2.10. Genetic Characterization of B. henselae

The *B. henselae* PCR-positive products from tick samples, tick feces, blood samples, and bacterial colonies were purified using a Plus DNA Clean/Extraction Kit (GMbiolab Co., Ltd., Taichung, Taiwan) and sent for nucleotide sequencing (Genomics BioScience and Technology Co., Ltd., Taiwan). Sequence data were compared with known sequences deposited in the GenBank database using the NCBI nucleotide BLAST tool. For genetic analysis, validated sequences were aligned and analyzed by using MegAlign (DNASTAR, Inc., Madison, WI, USA).

### 2.11. Identification of Bacterial Colonies Other Than B. henselae by Mass Spectrometry

The bacterial colonies that were negative for *B. henselae* detection in PCR were randomly selected and harvested, suspended in absolute ethanol, and sent to the Animal Health Research Institute, Council of Agriculture, Executive Yuan, Taiwan for classification and identification using matrix-assisted laser desorption/ionization time-of-flight mass spectrometry (MALDI-TOF MS) processing (Bruker Matrix HCCA, portioned). The raw spectra were analyzed by using the MALDI Biotyper 2.0 software (Bruker Daltonics, Leipzig, Germany). Two colonies, confirmed to be *B. henselae* by PCR from a stray cat in Taitung, Taiwan, were harvested to serve as a positive control. The microorganisms were identified according to the modified score values proposed by the manufacturer: a score value ≥ 2 indicated species identification, a score value between 1.7 and 1.9 indicated genus identification, and a score value < 1.7 indicated unreliable identification.

### 2.12. Statistical Analysis

Descriptive statistics was used to analyze the percentage of successfully engorged ticks and *B. henselae*-positive tick samples after taking blood meals. The tick attachment and engorgement rates in the control and experimental groups were calculated and compared using Fisher’s exact test; *p* < 0.05 was considered statistically significant (GraphPad Prism 8.4.2 software, San Diego, CA, USA).

## 3. Results

### 3.1. Rhipicephalus sanguineus s.l. Ticks Engorged by Artificial Membrane Feeding System

**Figure 2:**
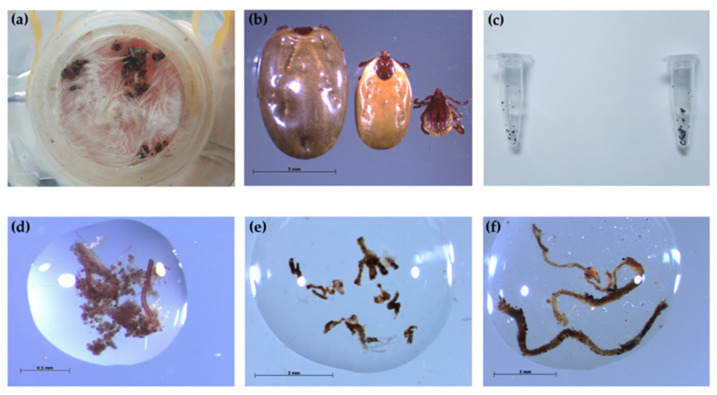
*Rhipicephalus sanguineus* s.l. adults engorged by artificial membrane feeding system and tick samples collected/dissected during and after feeding. (**a**) Ticks attached to the mouse skin; (**b**) engorged *R. sanguineus* s.l. female (left) and semi-engorged female (middle) and male (right); (**c**) pools of fecal samples collected from tick containers at 12 h (left) and 24 h (right) after the beginning of the experiment; (**d**) dissected salivary glands from semi-engorged ticks; (**e**) dissected midgut from semi-engorged ticks; (**f**) dissected ovaries from adult ticks.

Adult ticks started to attach to the mouse skins around 6 h after being placed in the feeders, and some ticks reattached after changing the mouse skins (Figure 2a). At the end of adult feeding, females spontaneously detached from the skins were considered engorged females, and those that were still attached to the mouse skins were considered semi-engorged females (Figure 2b). Tick feces was firstly found in the tick container around 12 h after the beginning of the experiment (Figure 2c). After 14 days of feeding, 16% (4/25) were fully engorged, and 56% (14/25) of females were semi-engorged in the experimental group. For the control group, 25% (5/20) and 50% (10/20) of ticks were fully engorged and semi-engorged females, respectively (Table 1). There was no statistically significant difference (*p* = 1.0000) between the attachment rate in the control group (75%) and the experimental group (72%).

### 3.2. Bartonella henselae Acquisition by Adult Ticks during Feeding on Infected Blood Meal

#### 3.2.1. *Bartonella henselae* Detection in *R. sanguineus* s.l. Adult Samples

After 14 days of blood feeding, DNA samples from dissected salivary glands, midguts, and ovaries were tested for *B. henselae* DNA presence by nested PCR (Figure 2d–f). In the experimental group, PCR results showed that 62.5% (5/8) of midguts and 37.5% (3/8) of salivary glands of male ticks harbored the *B. henselae* DNA fragment (Table 2). From semi-engorged females, 80% (8/10) of midguts, 60% (6/10) of salivary glands, and 30% (3/10) of carcasses were positive for *B. henselae* DNA detection, while the male carcasses and female ovaries were all PCR-negative (Table 2). In the control group, all samples were negative for *B. henselae* DNA detection (Table 2). All obtained sequences from midguts (*n* = 2) and salivary glands (*n* = 3) of male ticks, and midguts (*n* = 2), salivary glands (*n* = 3), and carcasses (*n* = 2) of female ticks showed 100% identity with the *B. henselae* sequence (accession number MT095055.1).

#### 3.2.2. Persistence of *B. henselae* in *R. sanguineus* s.l. Feces

**Figure 3:**
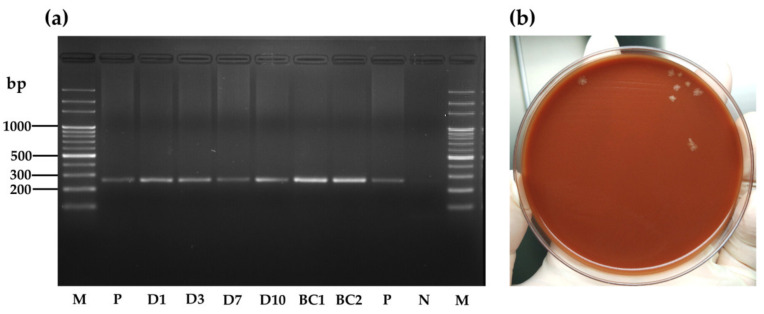
Detection of *B. henselae* by nested PCR (**a**) and culture (**b**) from fecal samples collected on day 14 and incubated for 1 day after *R. sanguineus* s.l. adult tick feeding on *B. henselae*-infected blood through artificial membrane feeding system. M, DNA marker; D1, fecal sample collected on day 14 and incubated for 1 day; D3, fecal sample collected on day 14 and incubated for 3 days; D7, fecal sample collected on day 14 and incubated for 7 days; D10, fecal sample collected on day 14 and incubated for 10 days; BC1 and BC2, bacterial colonies isolated from feces collected on day 14 and incubated for 1 day; P, positive control (*B. henselae* DNA); N, negative control (distilled water).

Among the 19 pooled fecal samples, all samples from the experimental group including four which were collected on day 14 and incubated for 1, 3, 7, and 10 days were positive for *B. henselae* PCR, except the samples collected at 24 h on day 1, on day 9, and on day 12 (Figure 3a). All fecal samples from the control group were PCR-negative. During an incubation period, the tick fecal sample was still fresh, sticky, and moist on day 1, while the feces on day 3, 7, and 10 were all dry. For *B. henselae* isolation, after 21 days of culture, bacterial colonies were observed only for pooled fecal samples, which were collected on day 14 and incubated for 1 day (Figure 3b). Two randomly selected bacterial colonies from the fecal samples on the agar plate showed amplification of *B. henselae* DNA after tested by PCR (Figure 3a). Moreover, viable *B. henselae* in this fecal sample was estimated to be 2.5 × 10^9^ CFU/mL. All obtained sequences from pooled fecal samples (*n* = 5) and bacterial colonies (*n* = 2) from culture showed 100% identity with the *B. henselae* isolate (accession number MT095055.1).

### 3.3. Absence of B. henselae DNA in Pooled Eggs and Unfed Larvae Derived from B. henselae-Infected Female Ticks

After the oviposition period (ranged 6–11 days), *B. henselae* DNA was detected in 33.3% (1/3) of salivary glands from females that laid eggs, but not in the midguts, ovaries, or tick carcasses (Table 2). Pooled egg samples laid by *B. henselae*-infected engorged females were all negative for *B. henselae* DNA detection. In this study, the duration of egg hatching and emergence of larvae ranged from 18–20 days. Pooled unfed larvae were all negative for *B. henselae* PCR. No *B. henselae* DNA was detected in samples from the control group.

### 3.4. Bartonella henselae Transmission by Larvae Infected at the Adult Stage

A total of 300 larvae from the experimental group and 250 larvae from the control group were separately fed with noninfected goat blood for 7 days. Engorged larvae started to detach from the mouse skin on day 5 of feeding. After 7 days of feeding, 17% (51/300) larvae were fully engorged, and 9.3% (28/300) were semi-engorged in the experimental group. For the control group, 15.6% (39/250) and 34% (85/250) of larvae were engorged and semi-engorged, respectively (Table 1). The attachment rate of larvae in the control group (49.6%) was significantly higher than that in the experimental group (26.3%) (*p* = 0.0001).

**Figure 4:**
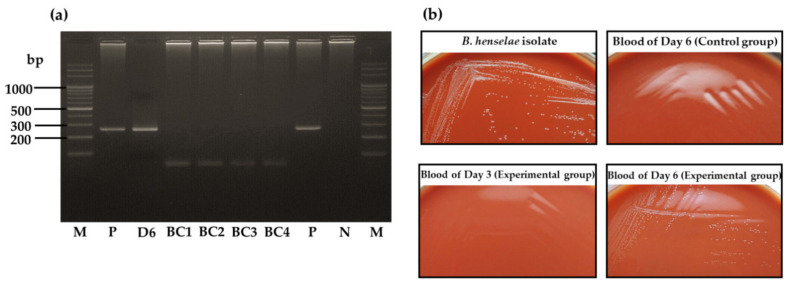
Detection of *B. henselae* DNA and isolation of *B. henselae* colonies from daily collected blood samples during feeding of larvae infected at the preceding adult stage through artificial membrane feeding system. (**a**) PCR results of blood sample and bacterial colonies from blood sample of day 6. M, DNA marker; D6, blood sample collected from feeder on day 6; BC1–4, bacterial colonies harvested from chocolate agar plate; P, positive control (*B. henselae* DNA); N, negative control (distilled water). (**b**) Isolation on chocolate agar of bacteria from blood samples during 7 days of larval feeding. After 14 days of culture, bacterial colonies of whitish–translucent color were observed only for the blood sample collected on day 6 in the experimental group. *B. henselae* isolate is shown as a positive control for comparison.

Blood samples were collected daily from feeders and tested for *B. henselae* DNA detection by PCR. *Bartonella henselae* DNA was detected only in the blood sample from day 6 of larval feeding from the experimental group (Figure 4a). The obtained sequence from the blood sample showed 100% identity with the *B. henselae* isolate (accession number MT095055.1). In the control group, blood samples were all PCR-negative. After larval feeding, no *B. henselae* DNA was detected from 10 pooled samples of engorged larvae from the experimental group or from the three pooled samples from the control group (results not shown).

The daily collected blood samples were also placed in Schneider *Drosophila* medium for 6 days, and then cultured on chocolate agar plates. After 10 days of culture, some colonies of whitish–translucent color were observed on the agar plate from the blood sample collected on day 6 from the experimental group, while no bacterial colonies were observed for other samples (Figure 4b). Four randomly selected colonies were all negative for *B. henselae* DNA detection by PCR (Figure 4a). By using MALDI-TOF MS, the results showed that the bacterial colonies had the best match with *Corynebacterium* spp., a tick gut microbiome member, with score values of 1.825, 1.833, 1.947, and 1.992.

## 4. Discussion

The present study was performed to validate *B. henselae* infection in *R. sanguineus* s.l. adult ticks and the transmission of the bacterium to their instars. The results obtained showed that *B. henselae* can be acquired by adult ticks when feeding on infected blood. We also demonstrated that larvae obtained from these adult females were able to inject some *B. henselae* DNA into blood during their feeding in the artificial feeding system. This evidence shows the possibility of transovarial transmission of *B. henselae* in *R. sanguineus* s.l. ticks. In addition, the isolation of *B. henselae* from adult tick feces emphasizes the possibility that tick feces could be a source of *B. henselae* transmission.

Indeed, we demonstrated that the artificial feeding system developed for *I. ricinus* [28] and previously validated to feed both larvae and nymphs of *R. sanguineus* s.l. [26] could be successfully applied on *R. sanguineus* s.l. adult feeding. Mouse skin with a thickness of around 300 µm was suitable for *R. sanguineus* s.l. adult feeding because the mouthparts of *R. sanguineus* s.l. are around 270 µm in males and 370 µm in females [32,33]. Because of its strength and thickness (around 1180 µm), rabbit skin was initially used to fed *I. ricinus* adults, which has a longer mouthpart (280 µm in males and 500 µm in females) than *R. sanguineus* s.l. and can maintain its strength and elasticity during the whole tick-feeding period of 21 days of *I. ricinus* [19,28,32]. However, when compared with rabbit skin, the mouse skin used here is thinner and degrades faster, which led us to renew the skin during the adult tick feeding process. According to our observation, some of semi-engorged *R. sanguineus* s.l. adults forcibly detached from the skin could reattach within 1 day of skin renewing and continue to feed on blood until the end of feeding.

The feeding process of ticks is slow and complex when compared to other blood-sucking arthropods [34]. It has been reported that the major tick-borne pathogens, such as *Borrelia* spp. and *Babesia* spp., can increase the tick feeding behavior in order to enhance pathogen acquisition and transmission [35,36,37,38,39,40,41]. In the present study, we observed the opposite finding regarding *B. henselae* infection in *R. sanguineus* s.l. The attachment rate of larvae in the experimental group was significantly lower than that in the control group. A similar finding was previously reported for *B. henselae* infection in *I. ricinus* ticks [42]. Such results were obtained through artificial feeding of ticks and not on naturally infected animals. It suggests that, for *B. henselae*, the presence of pathogens may directly affect the performance parameters of tick feeding rather than ticks responding indirectly to host cues of infection [34,42]. The selective adaptation of *Bartonella* spp. has been proposed among the wide range of arthropod vectors harboring various *Bartonella* spp., as some of them may not efficiently transmit the bacteria to their hosts [43]. A negative impact on the fitness of ticks suggests that ticks may not be the main vector of *B. henselae*.

*Bartonella henselae* can be experimentally transmitted by *I. ricinus* ticks through tick saliva [19]. During ixodid ticks feeding on the hosts, members of Kunitz family of serine protease inhibitors are secreted to disrupt the host angiogenesis and wound healing, as well as enhance blood uptake and digestion of the ticks, which is essential for tick feeding [44,45]. The *I. ricinus* serine protease inhibitor (*IrSPI*) was found to be the most overexpressed protein in tick salivary glands following *B. henselae* infection of *I. ricinus* [46]. It has been shown that the silencing of *IrSPI* can reduce tick feeding behavior and decrease *B. henselae* load in the tick salivary glands [46]. A further study on these inhibitors in *R. sanguineus* s.l. during *B. henselae* infection would be helpful to understand the interaction between *R. sanguineus* s.l. and *B. henselae* [44].

*Bartonella henselae* DNA was detected in midguts, salivary glands, and carcasses of both semi-engorged and engorged adult ticks that fed on *B. henselae*-infected blood. This finding emphasizes that *B. henselae* can be acquired by *R. sanguineus* s.l. ticks during blood meals, as hypothesized from previous epidemiological studies [17,24,25]. According to the positive PCR results of the dissected tick samples, our result demonstrated that the bacteria could survive in the tick and may, from the digestive tract, invade different tissues of the tick including the salivary glands. This suggests that *B. henselae* is able to resist the immune system of *R. sanguineus* s.l. as is the case for other tick-borne pathogens [36,47]. In this regard, in addition to the reported transmission by *I. ricinus* [19], it is noticeable that Billeter et al. reported that *B. henselae* could invade and replicate within *Amblyomma americanum*, *I. scapularis*, and *R. sanguineus* cell lines [48]. Further studies regarding the defense mechanisms of ticks during *B. henselae* infection should be performed for a better understanding of the interaction between the bacterium and tick and the bacterial viability in ticks.

In this study, *B. henselae* DNA was found in salivary glands of semi-engorged and engorged females at the end of feeding and after oviposition period, respectively. Although no *B. henselae* DNA was detected in eggs laid by infected females and newly hatched larvae, the bacterial DNA was detected in the blood sucked by those larval ticks, which suggests a potential of transovarial transmission. From the previous study regarding *B. henselae* infection in *I. ricinus* ticks, no *B. henselae* DNA were detected in salivary glands of nymphs (infected as larvae) and unfed females (infected as nymphs), but the bacterial DNA became detectable after those ticks took new blood meals [19]. Both findings suggested that blood refeeding of ticks could stimulate and multiply *B. henselae*, which then migrated to salivary glands and could transmit to the new host [19].

In cat fleas, *B. henselae* can survive in the gut for up to 9 days, while the bacterium can persist in flea feces for up to 12 days [49]. The long persistence of *B. henselae* in flea feces is necessary for *Bartonella* transmission among cats and from cats to humans [2,3,4]. In the present study, *B. henselae* was successfully isolated from *R. sanguineus* s.l. tick feces maintained at room temperature (24 °C, 60% humidity) for 1 day after tick feeding but not for those that remained 3, 7, or 10 days in the same conditions. This finding suggests that *B. henselae* can stay alive in tick midguts during the infectious blood meal and be shed through tick feces with limited persistence. The tick fecal sample was still fresh, sticky, and moist on day 1 of incubation, while the feces were all dry after that. In a previous study, Billeter et al. inoculated frozen *Bartonella* PCR-positive tick feces in dogs to investigate whether tick feces can be the source of *Bartonella* transmission [50]. The results showed that no dogs exhibited bacteremia, and using fresh tick feces instead of frozen ones was suggested by the authors [50]. According to these findings, it seems that time and environmental conditions, such as temperature, can affect the viability of *B. henselae* in tick feces and influence the bacterial transmission by feces. Although *B. henselae* persistence in tick feces is not long (at least 1 day), tick feces could still be a source of *B. henselae* transmission; however, this hypothesis still needs to be confirmed by further in vivo studies.

Although no *B. henselae* DNA was detected in the ovaries of semi-engorged females, pooled eggs, and unfed larvae, the presence of *B. henselae* DNA in blood sample during larval feeding suggests that *B. henselae* could remain in eggs and be transovarially transmitted to larvae, but maybe at an undetectable level in tick samples. In order to confirm the transovarial transmission, further experiments of *B. henselae* isolation from tick ovaries and larvae would need to be performed. Several tick-borne pathogens, such as *Babesia* spp. and *Rickettsia* spp., can be transmitted by transovarial transmission [51,52]. Those pathogens are probably taken into tick ovaries during vitellogenin protein transportation and fusion to form yolk granules in ovaries, which reach the highest concentration in fully engorged ticks [51,52,53].

*Bartonella henselae* DNA was detected in blood samples during larval feeding; however, no viable *B. henselae* could be successfully isolated. An explanation is that *R. sanguineus* s.l. larvae may transmit *B. henselae* to blood at a low concentration beyond the detection limit, since just a few microliters of tick saliva are injected into the blood during larval feeding [19]. In fact, even though the volume of blood used here was 10 times higher, it should be noted that, in the study of Cotté et al., *B. henselae* colonies were successfully isolated from blood samples following the feeding of nymphs and adult *I. ricinus* ticks that inject higher volume of saliva during feeding and, therefore, potentially more bacteria [19].

In the present study, a common tick microbiota *Corynebacterium* colonies was isolated on agar plates of blood culture from the experimental group. *Corynbacterium* spp. are predominant in the tick midgut microbiota of *Rhipicephalus* (*Boophilus*) *microplus*, *Haemophysalis* spp., and *Hyalomma* spp. ticks [54,55,56,57]. The identification of *Corynebacterium* colonies suggests that ticks can inject microbiota from their digestive tract into the blood and, therefore, potentially into the blood of vertebrate hosts during feeding. This has already been reported for *Coxiella*-like endosymbionts (*Coxiella*-LE), the common gut microbiota found in ixodid ticks, which can be transmitted to animal hosts via tick bites [58,59].

Our findings reinforce the possibility of *B. henselae* transmission by *R. sanguineus* s.l. ticks. The bacterial DNA could remain in the tick midguts and then be shed through tick feces. Its detection in other tick tissues such as salivary glands provides evidence of bacterial viability in the tick as it can move and colonize these tissues by crossing physiological barriers. Most importantly, bacterial DNA detected in the blood during feeding of progeny of infected females provides a vital clue on *B. henselae* transovarial transmission in *R. sanguineus* s.l. ticks and possible transmission to a host by larvae. Further studies, such as *B. henselae* isolation from tick samples, an in vivo study of *B. henselae*-infected tick feces, and protein analysis in tick salivary glands during *B. henselae* infection, should be performed for a better understanding of the interactions between *B. henselae* and ticks, as well as to clarify the vector competence of *R. sanguineus* s.l. and *B. henselae* transmission pathways.

## Figures and Tables

**Table 1:** Number of *R. sanguineus* s.l. females and larvae engorged on *B. henselae*-infected (experimental group) or noninfected (control group) goat blood though artificial membrane feeding system.

Ticks	*B. henselae*-Infected Group	Noninfected Group
Females (after 14 days of feeding)	*n* = 25	*n* = 20
No. of engorged ticks	4 (16.0%)	5 (25.0%)
No. of semi-engorged ticks	14 (56.0%)	10 (50.0%)
No. of unfed, inactive, or dead ticks	7 (28.0%)	5 (25.0%)
Attachment rate	18 (72.0%)	15 (75.0%)
Larvae (after 7 days of feeding)	*n* = 300	*n* = 250
No. of engorged ticks	51 (17.0%)	39 (15.6%)
No. of semi-engorged ticks	28 (9.3%)	85 (34.0%)
No. of unfed, inactive, or dead ticks	221 (73.6%)	126 (50.4%)
Attachment rate	79 (26.3%) *	124 (49.6%) *

Data were analyzed statistically to compare results between ticks fed on *B. henselae*-infected blood (experimental group) and noninfected blood (control group) by Fisher’s exact test (* *p* < 0.05).

**Table 2:** *Bartonella henselae* DNA detection from tick samples dissected at the end of adult feeding on *B. henselae*-infected or noninfected goat blood through artificial membrane feeding system and post oviposition.

Adult Ticks	No. of *B. henselae*-Positive Samples/No. of Tested Samples (%)	
	*B. henselae*-Infected Group	Noninfected Group
At the end of feeding		
Males (MG)	5/8 (62.5)	0/3 (0)
Males (SG)	3/8 (37.5)	0/3 (0)
Males (CC)	0/8 (0)	0/3 (0)
Females (MG)	8/10 (80)	0/3 (0)
Females (SG)	6/10 (60)	0/3 (0)
Females (OV)	0/10 (0)	0/3 (0)
Females (CC)	3/10 (30)	0/3 (0)
Post oviposition		
Females (MG)	0/3 (0)	0/2 (0)
Females (SG)	1/3 (33.3)	0/2 (0)
Females (OV)	0/3 (0)	0/2 (0)
Females (CC)	0/3 (0)	0/2 (0)

MG, midguts; SG, salivary glands; OV, ovaries; CC, carcasses.

## Data Availability

Not applicable.

## References

[1] Chomel B.B., Kasten R.W. (2010). Bartonellosis, an increasingly recognized zoonosis. J. Appl. Microbiol..

[2] Abbott R.C., Chomel B.B., Kasten R.W., Floyd-Hawkins K.A., Kikuchi Y., Koehler J.E., Pedersen N.C. (1997). Experimental and natural infection with Bartonella henselae in domestic cats. Comp. Immunol. Microbiol. Infect. Dis..

[3] Chomel B.B., Kasten R.W., Floyd-Hawkins K., Chi B., Yamamoto K., Roberts-Wilson J., Gurfield A.N., Abbott R.C., Pedersen N.C., Koehler J.E. (1996). Experimental transmission of Bartonella henselae by the cat flea. J. Clin. Microbiol..

[4] Foil L., Andress E., Freeland R.L., Roy A.F., Rutledge R., Triche P.C., O’Reilly K.L. (1998). Experimental infection of domestic cats with Bartonella henselae by inoculation of Ctenocephalides felis (Siphonaptera: Pulicidae) feces. J. Med. Entomol..

[5] Higgins J.A., Radulovic S., Jaworski D.C., Azad A.F. (1996). Acquisition of the cat scratch disease agent Bartonella henselae by cat fleas (Siphonaptera: Pulicidae). J. Med. Entomol..

[6] Iannino F., Salucci S., Di Provvido A., Paolini A., Ruggieri E. (2018). Bartonella infections in humans dogs and cats. Vet. Ital..

[7] Carithers H.A., Carithers C.M., Edwards R.O. (1969). Cat scratch disease: The larger view. Pediatrics.

[8] Arisoy E.S., Correa A.G., Wagner M.L., Kaplan S.L. (1999). Hepatosplenic cat-scratch disease in children: Selected clinical features and treatment. Clin. Infect. Dis..

[9] Chian C.A., Arrese J.E., Pierard G.E. (2002). Skin manifestations of Bartonella infections. Int. J. Dermatol..

[10] Chang C.C., Chomel B.B., Kasten R.W., Romano V., Tietze N. (2001). Molecular evidence of Bartonella spp. in questing adult Ixodes pacificus ticks in California. J. Clin. Microbiol..

[11] Chung C.Y., Kasten R.W., Paff S.M., Van Horn B.A., Vayssier-Taussat M., Boulouis H.J., Chomel B.B. (2004). Bartonella spp. DNA associated with biting flies from California. Emerg. Infect. Dis..

[12] Durden L.A., Ellis B.A., Banks C.W., Crowe J.D., Oliver J.H. (2004). Ectoparasites of gray squirrels in two different habitats and screening of selected ectoparasites for bartonellae. J. Parasitol..

[13] Rar V.A., Fomenko N.V., Dobrotvorsky A.K., Livanova N.N., Rudakova S.A., Fedorov E.G., Astanin V.B., Morozova O.V. (2005). Tickborne pathogen detection, Western Siberia, Russia. Emerg. Infect. Dis..

[14] Reeves W.K., Nelder M.P., Cobb K.D., Dasch G.A. (2006). *Bartonella* spp. in deer keds, *Lipoptena mazamae* (Diptera: Hippoboscidae), from Georgia and South Carolina, USA. J. Wildl. Dis..

[15] Reeves W.K., Szumlas D.E., Moriarity J.R., Loftis A.D., Abbassy M.M., Helmy I.M., Dasch G.A. (2006). Louse-borne bacterial pathogens in lice (Phthiraptera) of rodents and cattle from Egypt. J. Parasitol..

[16] Regier Y., Ballhorn W., Kempf V.A. (2017). Molecular detection of Bartonella henselae in 11 Ixodes ricinus ticks extracted from a single cat. Parasit. Vectors.

[17] Tsai Y.L., Lin C.C., Chomel B.B., Chuang S.T., Tsai K.H., Wu W.J., Huang C.G., Yu J.C., Sung M.H., Kass P.H. (2011). Bartonella infection in shelter cats and dogs and their ectoparasites. Vector Borne Zoonotic Dis..

[18] Lucey D., Dolan M.J., Moss C.W., Garcia M., Hollis D.G., Wegner S., Morgan G., Almeida R., Leong D., Greisen K.S. (1992). Relapsing illness due to Rochalimaea henselae in immunocompetent hosts: Implication for therapy and new epidemiological associations. Clin. Infect. Dis..

[19] Cotte V., Bonnet S., Le Rhun D., Le Naour E., Chauvin A., Boulouis H.J., Lecuelle B., Lilin T., Vayssier-Taussat M. (2008). Transmission of Bartonella henselae by Ixodes ricinus. Emerg. Infect. Dis..

[20] Reis C., Cote M., Le Rhun D., Lecuelle B., Levin M.L., Vayssier-Taussat M., Bonnet S.I. (2011). Vector competence of the tick Ixodes ricinus for transmission of Bartonella birtlesii. PLoS Negl. Trop. Dis..

[21] Krol N., Militzer N., Stobe E., Nijhof A.M., Pfeffer M., Kempf V.A.J., Obiegala A. (2021). Evaluating Transmission Paths for Three Different Bartonella spp. in Ixodes ricinus Ticks Using Artificial Feeding. Microorganisms.

[22] Dantas-Torres F. (2010). Biology and ecology of the brown dog tick, Rhipicephalus sanguineus. Parasites Vectors.

[23] Dantas-Torres F., Chomel B.B., Otranto D. (2012). Ticks and tick-borne diseases: A One Health perspective. Trends Parasitol..

[24] Satta G., Chisu V., Cabras P., Fois F., Masala G. (2011). Pathogens and symbionts in ticks: A survey on tick species distribution and presence of tick-transmitted micro-organisms in Sardinia, Italy. J. Med. Microbiol..

[25] Wikswo M.E., Hu R., Metzger M.E., Eremeeva M.E. (2007). Detection of Rickettsia rickettsii and Bartonella henselae in Rhipicephalus sanguineus ticks from California. J. Med. Entomol..

[26] Wechtaisong W., Bonnet S.I., Lien Y.Y., Chuang S.T., Tsai Y.L. (2020). Transmission of Bartonella henselae within Rhipicephalus sanguineus: Data on the Potential Vector Role of the Tick. PLoS Negl. Trop. Dis..

[27] Troughton D.R., Levin M.L. (2007). Life cycles of seven ixodid tick species (Acari: Ixodidae) under standardized laboratory conditions. J. Med. Entomol..

[28] Bonnet S., Jouglin M., Malandrin L., Becker C., Agoulon A., L’Hostis M., Chauvin A. (2007). Transstadial and transovarial persistence of Babesia divergens DNA in Ixodes ricinus ticks fed on infected blood in a new skin-feeding technique. Parasitology.

[29] Lejal E., Moutailler S., Simo L., Vayssier-Taussat M., Pollet T. (2019). Tick-borne pathogen detection in midgut and salivary glands of adult Ixodes ricinus. Parasites Vectors.

[30] Rolain J.M., Franc M., Davoust B., Raoult D. (2003). Molecular detection of Bartonella quintana, B. koehlerae, B. henselae, B. clarridgeiae, Rickettsia felis, and Wolbachia pipientis in cat fleas, France. Emerg. Infect. Dis..

[31] Sato S., Kabeya H., Negishi A., Tsujimoto H., Nishigaki K., Endo Y., Maruyama S. (2017). Molecular survey of Bartonella henselae and Bartonella clarridgeiae in pet cats across Japan by species-specific nested-PCR. Epidemiol. Infect..

[32] Krober T., Guerin P.M. (2007). In vitro feeding assays for hard ticks. Trends Parasitol..

[33] Wei J.C.J., Edwards G.A., Martin D.J., Huang H., Crichton M.L., Kendall M.A.F. (2017). Allometric scaling of skin thickness, elasticity, viscoelasticity to mass for micro-medical device translation: From mice, rats, rabbits, pigs to humans. Sci. Rep..

[34] Sojka D., Franta Z., Horn M., Caffrey C.R., Mares M., Kopacek P. (2013). New insights into the machinery of blood digestion by ticks. Trends Parasitol..

[35] Dai J., Narasimhan S., Zhang L., Liu L., Wang P., Fikrig E. (2010). Tick histamine release factor is critical for Ixodes scapularis engorgement and transmission of the lyme disease agent. PLoS Pathog..

[36] De la Fuente J., Antunes S., Bonnet S., Cabezas-Cruz A., Domingos A.G., Estrada-Pena A., Johnson N., Kocan K.M., Mansfield K.L., Nijhof A.M. (2017). Tick-Pathogen Interactions and Vector Competence: Identification of Molecular Drivers for Tick-Borne Diseases. Front. Cell. Infect. Microbiol..

[37] De la Fuente J., Villar M., Cabezas-Cruz A., Estrada-Pena A., Ayllon N., Alberdi P. (2016). Tick-Host-Pathogen Interactions: Conflict and Cooperation. PLoS Pathog..

[38] Hu R., Hyland K.E., Markowski D. (1997). Effects of Babesia microti infection on feeding pattern, engorged body weight, and molting rate of immature Ixodes scapularis (Acari: Ixodidae). J. Med. Entomol..

[39] Lefcort H., Durden L.A. (1996). The effect of infection with Lyme disease spirochetes (Borrelia burgdorferi) on the phototaxis, activity, and questing height of the tick vector Ixodes scapularis. Parasitology.

[40] Randolph S.E. (1991). The effect of Babesia microti on feeding and survival in its tick vector, Ixodes trianguliceps. Parasitology.

[41] Romashchenko A.V., Ratushnyak A.S., Zapara T.A., Tkachev S.E., Moshkin M.P. (2012). The correlation between tick (Ixodes persulcatus Sch.) questing behaviour and synganglion neuronal responses to odours. J. Insect Physiol..

[42] Liu X.Y., Cote M., Paul R.E., Bonnet S.I. (2014). Impact of feeding system and infection status of the blood meal on Ixodes ricinus feeding. Ticks Tick-Borne Dis..

[43] Tsai Y.L., Chang C.C., Chuang S.T., Chomel B.B. (2011). Bartonella species and their ectoparasites: Selective host adaptation or strain selection between the vector and the mammalian host?. Comp. Immunol. Microbiol. Infect. Dis..

[44] Blisnick A.A., Foulon T., Bonnet S.I. (2017). Serine Protease Inhibitors in Ticks: An Overview of Their Role in Tick Biology and Tick-Borne Pathogen Transmission. Front. Cell. Infect. Microbiol..

[45] Islam M.K., Tsuji N., Miyoshi T., Alim M.A., Huang X., Hatta T., Fujisaki K. (2009). The Kunitz-like modulatory protein haemangin is vital for hard tick blood-feeding success. PLoS Pathog..

[46] Liu X.Y., de la Fuente J., Cote M., Galindo R.C., Moutailler S., Vayssier-Taussat M., Bonnet S.I. (2014). IrSPI, a tick serine protease inhibitor involved in tick feeding and Bartonella henselae infection. PLoS Negl. Trop. Dis..

[47] Hajdusek O., Sima R., Ayllon N., Jalovecka M., Perner J., de la Fuente J., Kopacek P. (2013). Interaction of the tick immune system with transmitted pathogens. Front. Cell. Infect. Microbiol..

[48] Billeter S.A., Diniz P.P., Battisti J.M., Munderloh U.G., Breitschwerdt E.B., Levy M.G. (2009). Infection and replication of Bartonella species within a tick cell line. Exp. Appl. Acarol..

[49] Bouhsira E., Franc M., Boulouis H.J., Jacquiet P., Raymond-Letron I., Lienard E. (2013). Assessment of persistence of Bartonella henselae in Ctenocephalides felis. Appl. Environ. Microbiol..

[50] Billeter S.A., Kasten R.W., Killmaster L.F., Breitschwerdt E.B., Levin M.L., Levy M.G., Kosoy M.Y., Chomel B.B. (2012). Experimental infection by capillary tube feeding of Rhipicephalus sanguineus with Bartonella vinsonii subspecies berkhoffii. Comp. Immunol. Microbiol. Infect. Dis..

[51] Chauvin A., Moreau E., Bonnet S., Plantard O., Malandrin L. (2009). Babesia and its hosts: Adaptation to long-lasting interactions as a way to achieve efficient transmission. Vet. Res..

[52] Burgdorfer W., Brinton L.P. (1975). Mechanisms of transovarial infection of spotted fever Rickettsiae in ticks. Ann. N. Y. Acad. Sci..

[53] Rosell R., Coons L.B. (1992). The role of the fat body, midgut and ovary in vitellogenin production and vitellogenesis in the female tick, Dermacentor variabilis. Int. J. Parasitol..

[54] Andreotti R., Perez de Leon A.A., Dowd S.E., Guerrero F.D., Bendele K.G., Scoles G.A. (2011). Assessment of bacterial diversity in the cattle tick Rhipicephalus (Boophilus) microplus through tag-encoded pyrosequencing. BMC Microbiol..

[55] Perveen N., Muzaffar S.B., Vijayan R., Al-Deeb M.A. (2020). Microbial communities associated with the camel tick, Hyalomma dromedarii: 16S rRNA gene-based analysis. Sci. Rep..

[56] Karim S., Budachetri K., Mukherjee N., Williams J., Kausar A., Hassan M.J., Adamson S., Dowd S.E., Apanskevich D., Arijo A. (2017). A study of ticks and tick-borne livestock pathogens in Pakistan. PLoS Negl. Trop. Dis..

[57] Loong S.K., Lim F.S., Khoo J.J., Lee H.Y., Suntharalingam C., Ishak S.N., Mohd-Taib F.S., AbuBakar S. (2020). Culturable pathogenic bacteria in ticks parasitizing farm animals and rodents in Malaysia. Trop. Biomed..

[58] Duron O., Noel V., McCoy K.D., Bonazzi M., Sidi-Boumedine K., Morel O., Vavre F., Zenner L., Jourdain E., Durand P. (2015). The Recent Evolution of a Maternally-Inherited Endosymbiont of Ticks Led to the Emergence of the Q Fever Pathogen, Coxiella burnetii. PLoS Pathog..

[59] Tsementzi D., Castro Gordillo J., Mahagna M., Gottlieb Y., Konstantinidis K.T. (2018). Comparison of closely related, uncultivated Coxiella tick endosymbiont population genomes reveals clues about the mechanisms of symbiosis. Environ. Microbiol..

